# A hole lot of trouble: Severe bilateral anastomotic dehiscence following bilateral single sequential lung transplant

**DOI:** 10.1002/rcr2.1164

**Published:** 2023-05-23

**Authors:** Cameron White, Alice Stuart, Marshall Plit, Monique Malouf, Paul Lilburn

**Affiliations:** ^1^ Department of Medicine The Alfred, 55 Commercial Road Melbourne Victoria Australia; ^2^ Respiratory Department Prince of Wales Hospital Sydney New South Wales Australia; ^3^ School of Health Sciences University of New South Wales Sydney New South Wales Australia; ^4^ MQ Health Macquarie University Hospital Sydney New South Wales Australia

**Keywords:** airway complications, dehiscence, lung transplant

## Abstract

Airway complications post lung transplant including ischaemia and dehiscence have a significant associated mortality (2%–4%) and morbidity. We describe a case of a 22‐year‐old female who developed significant bilateral anastomotic dehiscence with severe ischaemia following a bilateral single sequential lung transplant (BSSLTx). Following an intensive antimicrobial regimen, judicious bronchoscopic surveillance, and a prolonged inpatient stay, the dehiscence resolved without requiring further surgical intervention. Our case highlights a space in the literature for further research with regard to airway complications post‐lung transplant and their management.

## Graphical abstract

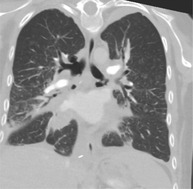

## INTRODUCTION

In 1963 the first human lung transplantation was performed.^1^ The patient died 18 days later secondary to renal failure and malnutrition.^2^ The next 15 years saw the majority of lung transplant recipients dying secondary to bronchial anastomotic complications.^3^ Presently, these airway complications occur in roughly 10%–20% of lung transplants and are associated with significant morbidity and mortality.^4^ In 2018, the International Society for Heart and Lung Transplantation (ISHLT) produced a consensus statement in regard to definitions and grading of airway complications post‐lung transplantation.^5^ Prior to this consensus statement, the variability in the definition of airways complications across sites made research in the area difficult. In this case, report we describe a 22‐year‐old female who developed significant bilateral anastomotic dehiscence with severe ischaemia following a bilateral single sequential lung transplant (BSSLTx) to treat neonatal bronchiolitis obliterans.

## CASE REPORT

A 22‐year‐old female with bronchiolitis obliterans and concomitant bronchiectasis was referred for a double lung transplant in 2016. The patient's condition deteriorated in 2014 with significant weight loss, decreased exercise tolerance, worsening productive cough, and an increasing frequency of hospital admissions for infections.

Lung function pre‐transplant demonstrated an FEV1 of 0.89 L (26% predicted), FVC of 2.42 L (61% predicted), TLC of 94% predicted, and DLCO of 59% predicted. Her past medical history was significant for combined central and obstructive sleep apnoea. This was treated with nocturnal CPAP at 9 cm H_2_O. Her baseline PCO_2_ ranged between 45 and 52 mmHg. There were no other comorbid diseases in her history or established during transplantation workup. Pre‐transplant sputum microbiology was significant for Methicillin sensitive *Staphylococcus aureus*, *Aspergillus fumigatus*, *Stenotrophomonas maltophilia*, and *Pseudomonas aeruginosa* (PsA).

The surgery occurred with no complications. There was severe adhesion between the native lung and pleura although the native lungs were extracted with no complication. The ventilation time prior to graft harvest was less than 72 h. There was roughly 36 h between clinical brain death and graft harvest. The donor was a female lifelong non‐smoker with nil respiratory background. There was a good size match between the donor and recipient. The donor received Ischaemic time of the left donor lung was 242 min. The ischaemic time of the right donor lung was 394 min. Our site has an ischaemic time target of 360 min or less.

Following the BSSLTx the patient was extubated on day 1 with primary graft dysfunction grade 0. Day 1 bronchoscopy demonstrated a normal appearance of both airway anastomoses with extensive clot that required suctioning with nil underlying bleeding evident. The anastomoses was described as healthy. Bronchial washings grew mucoid PsA. She was given standard immunosuppression as per the local guidelines; tacrolimus infusion (target trough levels of 12–15 ng/mL), IV methylprednisoloneand IV/PO mycophenolate 1500 mg BD. Initial empirical antimicrobial therapy was targeted to the patient's pre‐transplant microbiology and consisted of IV vancomycin, IV piperacillin‐tazobactam 4.5 g eight hourly, and IV voriconazole 200 mg twice daily. Standard prophylactic agents were commenced including azithromycin 250 mg PO three time weekly for bronchiolitis obliterans syndrome (BOS), Bactrim 800 mg/160 mg PO daily for *Pneumocystis jirovecii* and nebulised amphotericin B for fungal anastomotic breakdown.

Our patient made steady progress until day 4 postoperatively when tachycardia prompted an urgent computed tomography (CT) chest. This demonstrated a large right haemopneumothorax with collapse of the right middle lobe, and segmental collapse of the lower lobes bilaterally. An emergent re‐do thoracotomy for a right pleural cavity bleed was performed. The pleural fluid sample from this re‐do thoracotomy also grew mucoid PsA. Susceptibility analysis demonstrated resistance to piperacillin‐tazobactam and sensitivity to meropenem, tobramycin, and ceftazidime. As such her piperacillin‐tazobactam was replaced with meropenem 2 g eight hourly.

A bronchoscopy was performed day 7 post‐transplant for routine surveillance and demonstrated normal healing anastomoses bilaterally. Washings again grew mucoid PsA which was now resistant to piperacillin‐tazobactam and our patient was continued on meropenem. As per local guidelines the next surveillance bronchoscopy would be performed 21 days post‐transplant and would include transbronchial biopsies to assess for cell mediated rejection.

**FIGURE 1:**
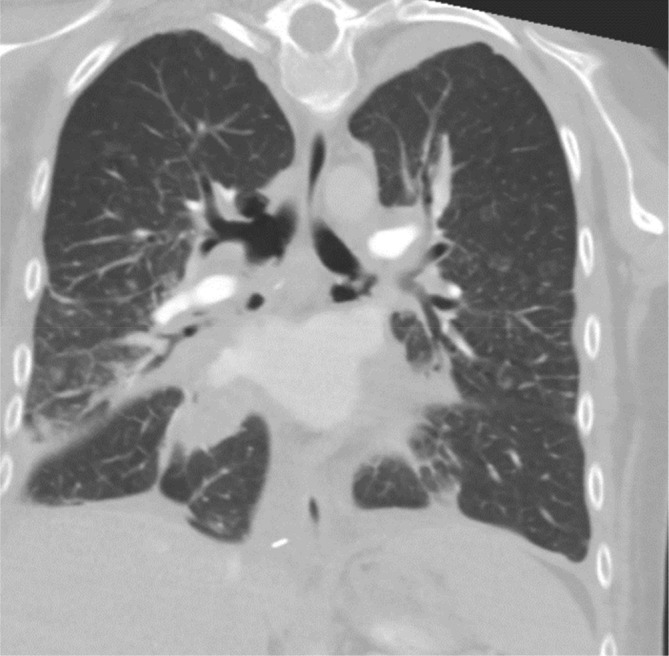
Day 18 postoperatively — CT Chest demonstrating large right sided bronchocele.

Our patient continued to progress well following her re‐do thoracotomy with improving inflammatory markers, sputum volume, exercise tolerance, and decreasing oxygen requirements. Day 18 post‐transplant she went into a supraventricular tachycardia (SVT) which resolved with a valsalva manoeuvre. To investigate the SVT a CT pulmonary angiogram (CTPA) was performed same‐day which excluded a pulmonary embolism. The CTPA demonstrated multiple locules of mediastinal gas which communicated freely with the airways and widespread peribronchovascular soft tissue thickening with areas of bronchial stenosis and plugging bilaterally (see Figure 1).

**FIGURE 2:**
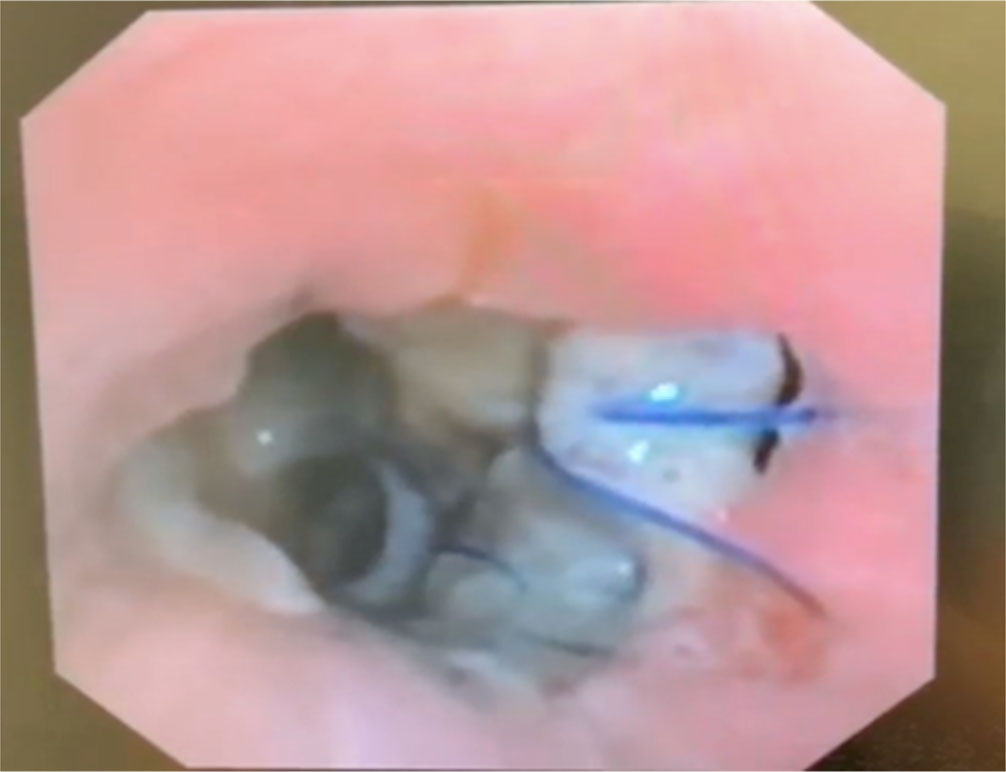
Left main bronchi. Day 18 Bronchoscopy, left anastomotic dehiscence and adjacent mucosal defect.

An urgent bronchoscopy was arranged that day which demonstrated severe bilateral anastomotic ischaemic changes with dehiscence. There was a large defect in the left anastomosis large enough for the left pulmonary artery to be directly visualized endobronchially, see Figure 2. On the right there was a large ischaemic out pouching distal to right anastomosis but proximal to the right upper lobe orifice. There was a thin‐walled, mobile defect in right main bronchus. There were purulent secretions throughout and bronchial washings were collected.

Following these findings, IV vancomycin and nebulised tobramycin were added to the antimicrobial regimen. Given concern for infection and high levels the tacrolimus dose was reduced. The patient and her family were informed of the bronchoscopy findings and counselled on the risks including life‐threatening airway bleeding, further anastamotic dehiscence, graft failure, and potentially death. It was decided that conservative management would be pursued with aggressive control of infection and avoiding further ischaemic insults. Staff involved in her care were educated on these risk also. Psychosocial support was provided to the patient, her family, and healthcare workers.

The cardiothoracic surgical team reviewed the case and felt given the level of ischaemic change at the anastomosis surgical intervention at present was not indicated due to the risk of causing further anastamotic damage. There was also concern surgical intervention may cause break down of the right sided bronchocoele (seen in Figure 1) causing a bronchopleural fistula.

Another bronchoscopy was performed day 28 post‐transplant to monitor the anastamoses. On the left side there was mild improvement of the anastomotic ischaemia, however, it remained severe. The dehiscence in the anterior portion persisted. Similarly on the right, the anastomotic ischaemia and adjacent defect had marginally improved. There was extensive mucus plugging which was aspirated.

A repeat CT performed on day 32 post‐transplant was performed for ongoing monitoring and demonstrated the left and right main bronchi defects were stable. There was ongoing communication with the mediastinum however the right sided pneumomediastinum had resolved.

**FIGURE 3:**
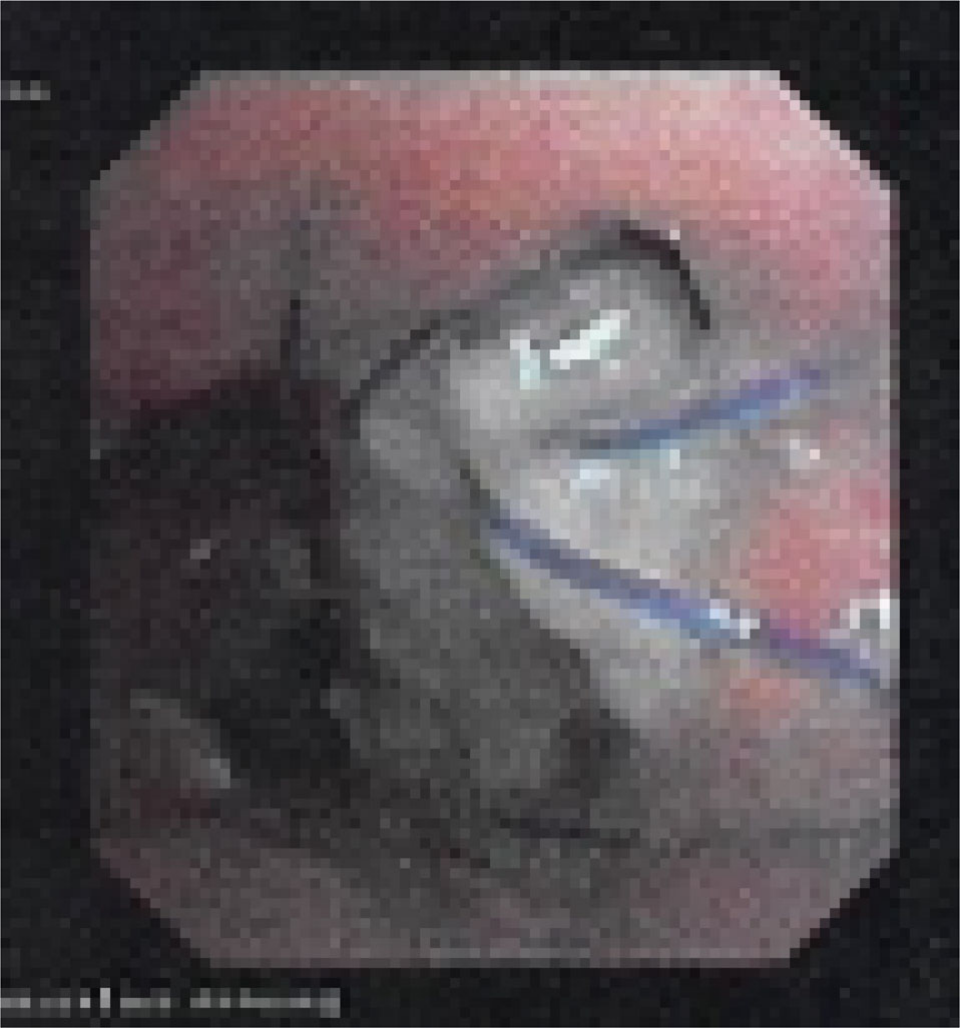
Left main bronchi. Day 39 bronchoscopy, left anastomotic ischaemia, dehiscence and adjacent hole. Improving mildly.

Ongoing monitoring of the anastomotic defects at day 39 performed with CT and bronchoscopy which demonstrated a mild reduction in the size of the defects and improvement in degree of pneumomediastinum but ongoing ischaemia bilaterally (see Figure 3). Bronchoscopy at day 52 showed further mild improvement in the dehiscence and stable ischaemia.

Throughout this time our patient remained on intensive antibiotic therapy and bronchoscopy washings had no further microbiological growth of significance. Inflammatory markers remained low and our patient remained afebrile. Immunosuppressive therapy was slowly re‐uptitrated and remained in the therapeutic range. She remained well and was successfully rehabilitated in the acute medical ward.

Our patient was discharged home on day 50 post‐transplant. On discharge the vancomycin was ceased; the meropenem was changed to piperacillin‐tazobactam via continuous infusion; nebulised vancomycin was continued; doxycycline 100 mg twice daily was added. All other standard post‐transplant medications were continued.

A bronchoscopy on day 57 was performed as an outpatient and demonstrated significant improvement in bilateral anastomotic ischaemia and adjacent dehiscence. The intravenous antimicrobials were ceased days 86 days post‐transplant following a bronchoscopy with nil microbiological growth. A CT scan performed 5 months post‐transplant has confirmed the successful resolution of the anastomotic dehiscence and all related changes. The patient remains well.

## DISCUSSION

Airway complications continue to be a source of morbidity and mortality early on post lung transplantation.^2^ In 1970, a review of 23 lung transplantations by 20 different surgeons revealed 13 of those developed noteworthy airway complications.^2^ Since, there have been revisions and improvements in; anastomotic technique, organ preservation, donor management, and post‐operative medical management.

Airway complications can include; ischaemia and necrosis, dehiscence, stenosis, malacia, and infectious. Mortality is 2%–4%.^4^ Morbidity is significant, leading to a poorer quality of life arising from the increased symptom burden and the need for repeated procedures.^4^ Thirty‐five percent of those with a previously treated airway complication will experience a second and 70% of those will experience a third.^4^ Untreated airway complications result in worse early survival compared to treated—62% versus 82% at 12 months.^4^

Anastomotic ischaemia and necrosis is part of the normal early healing phase but should resolve within the first 2–3 weeks post‐transplant.^4^ More severe ischaemia remains a risk factor for dehiscence.^4^ Bronchial artery anastomosis is not performed as standard during lung transplant and remains a key contributor to early large airway ischaemia.^4^

Dehiscence is considered the splitting of the anastomosis and can be partial or complete.^6^, ^7^ Whilst incidence ranges from one to 10% it carries a high mortality rate necessitating a surveillance program.^6^, ^7^ CT detection is accurate with a reported 100% sensitivity and 94% specificity.^5^ CT can detect per‐bronchial air, wall defects, and dissection into the mediastinum.^5^ Despite this, direct visualization with bronchoscopy still remains the gold standard of diagnosis.^5^, ^6^ Most transplant units utilize bronchoscopic surveillance with simultaneous surveillance for cell mediated rejection with transbronchial biopsies.^5^

Risk factors for airway necrosis and dehiscence, as seen in our patient, can be grouped into pre and post‐transplant as outlined in the Table 1.^5^, ^8^

**TABLE 1:** Risk factors for airway necrosis and dehiscence grouped into pre and post‐transplant.

Pre‐transplant	Post‐transplant
Prolonged mechanical ventilation of the donor recipient	Prolonged mechanical ventilation
Size matching	Primary graft dysfunction
Organ preservation	Hypoperfusion
Microbiological contamination	Microbiological contamination
Ischaemic time	Immunosuppression
Surgical technique	Acute cellular rejection (ACR)

Organ preservation, procurement techniques are relevant for example, healing can be compromised if the airways are improperly manipulated or preserved prior to implantation.^9^ This is because endothelial oedema and reperfusion injury decrease retrograde bronchial perfusion thus increasing the risk of ischaemia.^9^ Other organ preservation techniques that can be considered are the choice of perfusate, glucose solutions extend time to up to 24 h in canine model, and the technique of solution administration, studies show superior results with antegrade and retrograde routes.^9^ In addition, prostaglandin E1 has been shown to extend preservation times to 22–24 h, improving perfusate distribution by pulmonary vasodilation.^9^

Primary graft dysfunction leads to increased airway ischaemia secondary to interstitial oedema and reduction in pulmonary blood flow.^7^, ^11^ Secondary increased vascular permeability demands a higher positive end‐expiratory pressure (PEEP) which furthers anastomotic stress.^11^

Whilst infection remains a significant risk factor for dehiscence its mechanism remains unclear.^5^, ^8^, ^10^ Both pre‐ and post‐transplant colonization in the recipient have been shown to be risk factors.^8^ It is inferred that localized infection from colonized organisms may compromise the integrity of the mucosa and promote ischaemia and necrosis.^10^ This highlights the importance of inhaled antimicrobials.^8^

With regards to our patient, relevant risk factors for her anastamotic ischaemia, necrosis, and subsequent dehiscence include hypoperfusion (during hypotensive episodes secondary to the intrapleural bleed) and significant infection. It is fortunate that other risk factors were minimal given the severity of the dehiscence. There was short donor time ventilation, adequate ischaemic time, no intra‐operative complications, good size, and HLA matching, no primary graft dysfunction and short post‐operative mechanical ventilation. Interestingly the anastomoses appeared well healing at the day 7 surveillance bronchoscopy and there were little to no clinical signs of dehiscence until the CTPA performed on day 18. On review of the case there was no clinical indicators that would have prompted the team to have performed a bronchoscopy earlier than the standard day 21.

The recently proposed grading system for airway complications by the ISHLT was not utilized in the bronchoscopic descriptions of our patients necrosis and dehiscence.^5^ The proposed system considers the location and extent of necrosis, location and extent of dehiscence, location and extent of stenosis and the location of malacia. The utilization of this tool would not have likely changed our patients outcome. However, consistency in reporting of the anastomotic changes would have made interbronchoscopic comparisons more accurate.

The treatment of anastomotic dehiscence is not well described in the literature and is dependent on severity and other patient comorbidities.^5^ Options generally include conservative, stenting or operative.^5^, ^8^, ^10^, ^11^, ^12^, ^13^ A number of case reports have described the use of stents, either silicone or metal.^5^, ^10^, ^11^, ^12^ Kapoor et al. concluded stenting proved a promising palliative option in those not suitable for further intervention following a case series of three.^10^ Chhajed et al. described two cases in which a metal stent was used which did not successfully control the dehiscence.^12^ Evidence supporting a conservative approach is limited to case reports with aggressive antimicrobial therapy and serial bronchoscopies.^5^, ^13^ Given the small patient numbers and unique patient situations more rigorous research and studies into optimal management of airway dehiscence remain difficult. Our case adds to the literature supportive a conservative approach as a viable long‐term option. Ideally, future research into the optimal management of airway complications would include randomized controlled trials.

## CONCLUSION

Our report described a case of severe bilateral airway anastomotic dehiscence post BSSLTx with a successful outcome with a conservative approach. Whilst each case of airway complication is unique this demonstrates when suitable, aggressive management of risk factors, and a non‐interventional watch and wait approach with close monitoring is reasonable.

Airway complications post‐lung transplantation remain a serious cause of early morbidity and mortality. Current literature is lacking in the optimal management of these complications. Despite the recent proposal of an international grading system the uptake of this system remains varied. Furthermore, its use in guiding clinical decision‐making remains unclear.

## AUTHOR CONTRIBUTIONS

**Paul Lilburn**: Lead and corresponding author. Directly involved in the conception of the manuscript, performed the literature review and critically appraised the articles selected; revised the manuscript for critically important intellectual content. Directly involved in the final approval of the version to be published. **Alice Stuart**: Co‐first author. Directly involved with the production of the manuscript and detailing the case described; drafted the work and revised the manuscript in conjunction with other co‐authors. Directly involved with the final approval of the version to be published. **Cameron White**: Co‐first author. Directly involved with the production of the manuscript and detailing the case described; drafted the work and revised the manuscript in conjunction with other co‐authors. Directly involved with the final approval of the version to be published. **Marshall Plit**: Involved in revising the manuscript for critically important intellectual content. Directly involved in the final approval of the version to be published. **Monique Malouf**: Involved in revising the manuscript for critically important intellectual content. Directly involved in the final approval of the version to be published.

## CONFLICT OF INTEREST STATEMENT

None declared.

## ETHICS STATEMENT

The authors declare that appropriate written informed consent was obtained for the publication of this manuscript and accompanying images.

## Data Availability

The data that support the findings of this study are available on request from the corresponding author. The data are not publicly available due to privacy or ethical restrictions.
